# Microwave De-Icing Efficiency Improvement of Asphalt Mixture with Structural Layer Optimization and Heat-Resistance Design

**DOI:** 10.3390/ma17133112

**Published:** 2024-06-25

**Authors:** Haibao Zhang, Xiaowei Zhou, Haoyan Guo, Ting Zhang, Xin Zhao, Zhenjun Wang

**Affiliations:** 1 School of Materials Science and Engineering, Chang’an University, Xi’an 710061, China; haibaozhang@chd.edu.cn (H.Z.); xwzhou@chd.edu.cn (X.Z.); zhangting@chd.edu.cn (T.Z.); zjwang@chd.edu.cn (Z.W.); 2 Xi’an Sipai New Materials Technology Co., Ltd., Xi’an 710608, China; 3 Shaanxi Union Research Center of University and Enterprise for Advanced Transportation Infrastructure Materials, Xi’an 710061, China; 4 Shaanxi Provincial Communications Planning & Design Institute, Xi’an 710075, China; xinzhao710065@163.com; 5 Xi’an Key Laboratory of Modern Transportation Function Materials, Xi’an 710064, China

**Keywords:** asphalt mixture, expanded perlite powder (EP), structural layer optimization, heat-resistance design, microwave de-icing efficiency

## Abstract

The application of microwave de-icing technology in road engineering is constrained by its low energy utilization rate, which can be attributed to low heat production rates and ineffective heat dissipation to the underlying pavement. In this work, asphalt mixtures are designed as an upper layer (heating layer) and a lower layer (thermal-resistance layer). Magnetite slag was selected as a microwave-sensitive source for generating heat, and expanded perlite powder was incorporated into the lower layer as a thermal resistance material. Structural layer optimization and thermal-resistance layer design of the asphalt mixture were carried out by changing the thickness of the upper and lower layers to further improve the heat production rates. The design effectiveness is comprehensively evaluated by factors such as the changing law of the average surface temperature of mixtures, ice-melting time, and cost-effectiveness analyses. The results show that EP possesses better thermal stability, lower microwave energy conversion ability and more excellent heat-resistance potential compared with mineral powder. The heat-resistance layer with EP can prevent heat from being conducted to the lower layer and promote it to concentrate on the specimen surface, which can endow the microwave heating efficiency of specimens to be further improved by up to 26.97% and the de-icing time reduced by 10%, ascribed to the heat-resistance design. Furthermore, the collaborative design of the structural layer optimization and heat-resistance layer can increase energy utilization efficiency and save microwave-absorbing materials while ensuring excellent microwave de-icing efficiency.

## 1. Introduction

A frozen sticky layer tends to form between the road surface and the snow/ice in winter, which makes it more difficult to remove ice or snow quickly. The adhesion strength between material and ice is related to many factors, such as the physical and chemical properties or the structural depth of solid material surfaces, the purity/salinity of water and the temperature [1]. At present, researchers have developed various active and passive de-icing methods, devices and maintenance strategies from various perspectives of machinery, physics and chemistry [2,3,4,5]. Excellent road ice/snow removal technology needs to meet the following requirements: (1) high efficiency, (2) environmentally friendly, (3) low cost and (4) high resource utilization [6].

Microwave heating technology meets all of the above conditions and can quickly eliminate the frozen layer by increasing the road’s average surface temperature in winter [7], which is achieved by radiating the microwave-absorbing materials that replace traditional road materials [8,9]. Microwave-absorbing materials mainly include carbonaceous materials or metal-containing materials, etc., all of which have good electromagnetic properties [10]. Magnetite slag (MS) is an industrial by-product that has been extensively utilized in pavement engineering and is attributed to its better engineering properties and functional role in microwave heating [11], which can help solve waste accumulation, avoid consequent environmental contamination and reduce the consumption of natural aggregates. One of the key factors for the excellent electromagnetic properties of magnetite is the fact that the metal content contained within it significantly reduces its electrical resistance properties, and the increase in conductivity gives it good microwave-absorption efficiency [12]. In addition, the incorporation of magnetite can improve some of the properties of asphalt mixtures; for example, in terms of stiffness, the stiffness of the mixtures of magnetite and granite and magnetite and gravel were increased by 45% and 32%, respectively [13].

The application of microwave heating technology in pavement engineering can achieve different purposes, such as induction heating and the self-healing of cracks [14,15], as well as improving the early strength of the pavement [16,17], production and recycling of road pavement materials [18] and microwave de-icing [10,19]. The design of microwave-heated pavement is determined by the final purpose. The common point of the above-mentioned purposes, other than microwave de-icing, is that it tends to provide sufficient heat to the pavement structure or complete material. The purpose of microwave de-icing technology places more emphasis on promoting the pavement surface to acquire enough heat to melt the frozen sticky layer. The accumulative heat on the pavement’s surface can be mainly guaranteed by two aspects: (1) sufficient heat production efficiency and (2) a higher heat utilization rate [20,21]. The microwave-absorbing and heating material used in this work is magnetite slag, whose microwave heating efficiency is five times that of limestone aggregates [22].

The heat utilization rate is related to the location of heat generation, conductivity and ambient temperature. A complete asphalt pavement includes the surface, base, subbase and cushion from top to bottom. The position of the microwave heating material should be as close as possible to the surface layer structure to enhance the utilization rate of the heat generated by the microwave. This is because microwaves have a certain degree of penetration and decrease with depth [12]. And the internal heat cannot be transmitted to the road surface in time, which can decrease the energy utilization due to the low thermal conductivity of mixtures [23]. Readjusting the mixture thickness can reduce the path for the heat generated inside to transmit to the surface [22], and better de-icing efficiency can be obtained [24]. However, consistently decreasing the thickness of the heating layer will not be sufficient to generate sustained heat to prevent re-icing prior to de-icing. In addition, the techniques for preventing the downward conduction of heat inside specimens have concentrated insufficient attention on the microwave de-icing area. At present, the main thermal insulation materials commonly used in asphalt mixtures are ceramics, ceramics, glass beads, expanded perlite powder (EP), etc. Among them, EP has been used in thermal insulation materials for decades due to its excellent thermal, acoustic and fire resistance properties [25]. Furthermore, the microwave-absorption performance of the specimen is closely related to the thickness of the heating layer containing microwave-absorbing materials [26]. In summary, the synergistic design of the upper heating layer and the lower heat resistance layer becomes a key issue to be addressed in this work.

Based on the above, asphalt mixtures containing different thicknesses of heating and heat barrier layers were designed in this work. The microwave heating efficiency, heat conduction and de-icing efficiency of mixtures were investigated. The heat accumulation and de-icing efficiency improvement mechanisms were discussed simultaneously. The achievement is in line with the actual needs of winter road management and is of great significance for ensuring driving safety in winter, improving road microwave de-icing technology and increasing the energy utilization rate.

## 2. Experimental Programs

### 2.1. Raw Materials

In this work, expanded perlite powder (EP) was adopted as the thermal-resistance material. EP was dried and pulverized by ball milling and then sieved by 0.075 mm to obtain the ground EP powder. The bulk density of the EP was 2.326 g/cm^3^. Mineral powder (MP) was used as a control.

Magnetite slag and limestone aggregate were used as aggregates; their properties were measured based on the Chinese specification (JTG E42-2005) [27], and the results are listed in Table 1. Only 4.75 mm–9.5 mm MS aggregates were incorporated into the mixtures as microwave-absorbing material due to their higher microwave heating efficiency [22]. The properties of asphalt are shown in Table 2.

### 2.2. Sample Preparation

**Figure 1:**
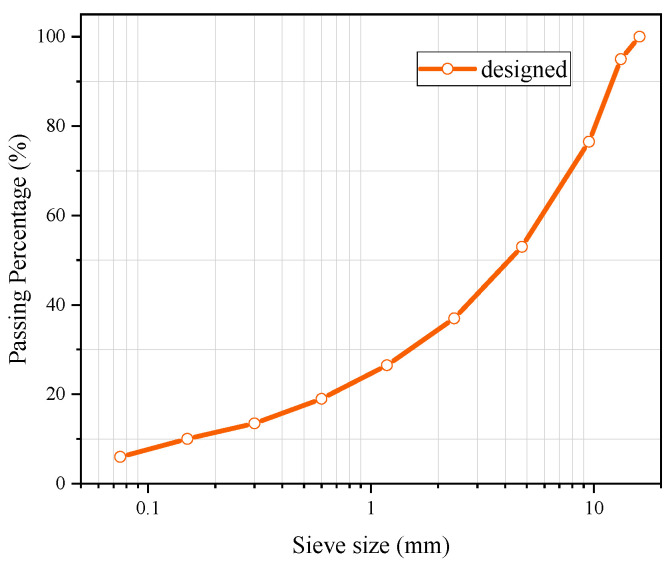
Aggregate gradation curve for asphalt mixture.

**Figure 2:**
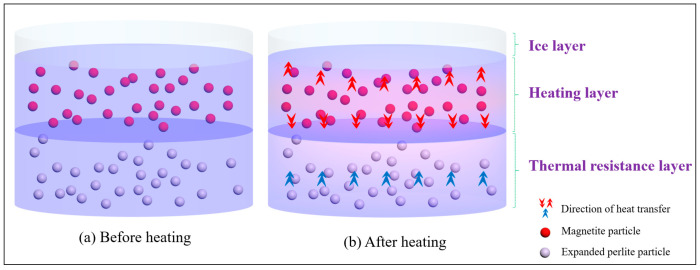
Schematic diagram for specimens with heat-resistance design.

The aggregate grading used for the asphalt mixture in this work is shown in Figure 1. Four kinds of asphalt mixtures were designed (including three experimental groups and one control group). The dimensions of the Marshall specimens of the experimental groups and the control group are Φ 63.5 mm × 101.6 mm. All three experimental groups contained an upper heating layer and a lower heat-resistance layer, as shown in Figure 2. The three experimental groups of specimens with a heat-resistance layer (HRS) are named according to the height of the heat-resistance layer: HRS G1 (30% of 63.5 mm, 19.0 mm), HRS G2 (50% of 63.5 mm, 31.7 mm) and HRS G3 (70% of 63.5 mm, 44.5 mm). The control group only contains one whole heating layer (4.75 mm–9.5 mm MS aggregates replaced the limestone aggregates with the same size by volume; the same applies below). The schematic diagram of the Marshall specimen’s structure is depicted in Figure 2. In Figure 2, the heat generated in the heating layer has a tendency to be transferred to both the ice layer and the heat resistance layer due to the temperature differences between different layers, and the thermal-resistance layer can block the heat transfer down and keep more heat in the heating layer.

The mixing proportion for the specimens is displayed in Table 3. Part of the results of our previous work [22] have been published and were compared and analyzed in this work to determine the effect of the heat-resistance layer. The asphalt mixture designed in the previous work also adopted a similar structural layer design. The difference is that the heat resistance materials were not introduced into the lower layer. The three experimental groups of specimens with the layered structure only (LSS) in the previous work are named LSS #1, LSS #2 and LSS #3, respectively, which correspond to HRS G3, HRS G2 and HRS G1 in this work.

The preparation process of the Marshall specimens in the experimental groups was divided into three steps: mixing of the upper and lower layers of asphalt mixture, layered filling and one-time compaction molding. The thermal-resistance effect of the heat-resistance layer is provided by the EP powder that replaces the MP with an equal volume. The optimal asphalt/aggregate ratio of the upper heating layer and the lower heat-resistance layer are 4.7% and 4.5% based on the Marshall test, respectively.

### 2.3. Methods

#### 2.3.1. Thermal-Resistance Potentiality Tests of EP

Expanded perlite powder was adopted in this work as the thermal-resistance material. Its thermal-resistance potential causes, as well as its thermal stability and microwave conversion ability have been examined in detail for feasibility verification before it was applied to the hot-mix asphalt mixture. The EP was firstly dried and pulverized by ball milling, and then sieved by 0.075 mm to obtain the ground EP powder with a bulk density of 2.326 g/cm^3^. The MP and MS powders were also prepared and compared after dry treatments (<0.075 mm) for 30 min at 105 °C to guarantee the expulsion of water in the samples.

The microstructures of the EP and MS powders were observed by an S-4800 environmental scanning electron microscope (SEM). In addition, map-scanning using an Electron Probe Micro Analyzer was also applied to characterize the compositions of particle surfaces. All samples were fixed on the conductive adhesive and sprayed with gold for 120 s to improve their conductivity.

The thermal stability of the EP powder was measured by thermogravimetric analysis (TA SDT 650, TA Instruments™, New Castle, DE, America). The temperature test range was 20–900 °C, the heating ramp was 20 °C/min, and the test was completed in a nitrogen atmosphere.

In addition, microwaves with a certain degree of penetration may still act on the EP in the underlying structure of the pavement. Therefore, the microwave heating efficiencies of the EP, MP and MS powders after 180 s of microwave radiation at room temperature were tested. The microwave radiation device is a microwave oven with an 800 W electromagnetic wave power and a 2.45 GHz frequency. The microwave radiation time of each sample was 180 s, and the temperature data were acquired by the Fluke TiS 75 thermal imaging camera (Fluke, Everett, WA, America) at 30 s intervals.

#### 2.3.2. Heat Generation and Conduction Test on Specimen Surface

The microwave heating efficiencies of specimens were first tested in this work. The side and bottom of the specimens were covered with aluminum foil to isolate the extra microwave, and then the specimens were placed in the microwave oven for radiation under different initial temperatures of −5 °C or −20 °C.

The heat on the specimen surface can be conducted until it is evenly dispersed within a period after microwave radiation. Therefore, the heat conduction state within 20 min after the microwave radiation ends was also detected in this work. Specimens were tested and photographed with an infrared thermal imager every 1 min under room temperature and windless conditions.

#### 2.3.3. Heat Conduction Test of Specimens In-Depth Direction

The heat generated by microwave radiation should be mainly dissipated on a road’s surface in the de-icing process, but a part of the heat can transmit to the lower structure, thus causing heat loss. This part of the work reveals whether the heat-resistance design plays a role in preventing heat conduction from the heating layer to the underlying structure by studying the temperature change rule in the depth direction of microwave radiation. The test process was similar to our previous work [22].

#### 2.3.4. Microwave De-Icing Time Test

**Figure 3:**
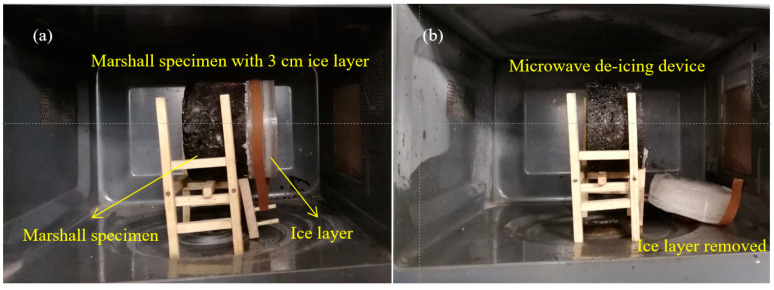
Microwave de-icing process: (**a**) before de-icing; (**b**) after de-icing.

The purpose of structural layer optimization or material composition design of asphalt mixtures ultimately lies in improving the de-icing efficiency. In this work, a laboratory device was designed to measure the de-icing time of specimens, and the microwave de-icing process is exhibited in Figure 3. The icing/de-icing device/process of the ice layer above the specimen is referred to in our previous work [22]. The initial temperature of the experiment is −5 °C and −20 °C, the ice layer thickness is 30 mm, and the de-icing time is accurate to 1 s.

## 3. Results

### 3.1. Feasibility Verification of Thermal-Resistance Materials

**Figure 4:**
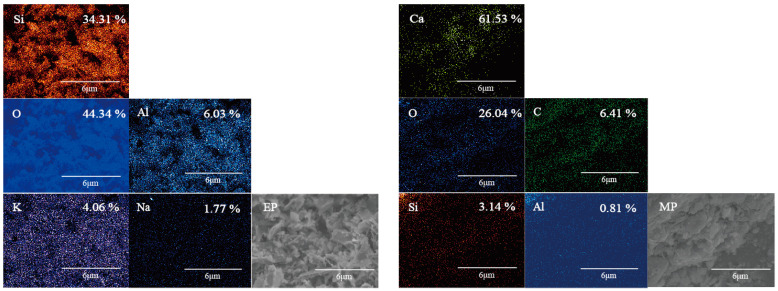
Compositional map-scanning of EP and MP surfaces and SEM/EDS results.

Figure 4 shows the morphology and composition scanning results of the EP and MP. From the perspective of morphology, there are a small amount of protrusions appearing on the MP particles’ surfaces. The grinding process transforms the morphology of the EP from a spherical porous shape to a broken flake structure. A huge number of fragments can be dispersed in the mixture to play a better role in heat resistance. From the perspective of chemical composition, the main chemical elements contained in the surfaces of EP and MP particles are evenly distributed, but there are differences in the mass percentages. The EP contains more Si elements compared with the MP, and the thermal conductivity of silicon dioxide is much lower than that of calcium dioxide [28]. Therefore, the EP has a more obvious advantage than the MP in terms of heat resistance from the perspective of chemical composition.

**Figure 5:**
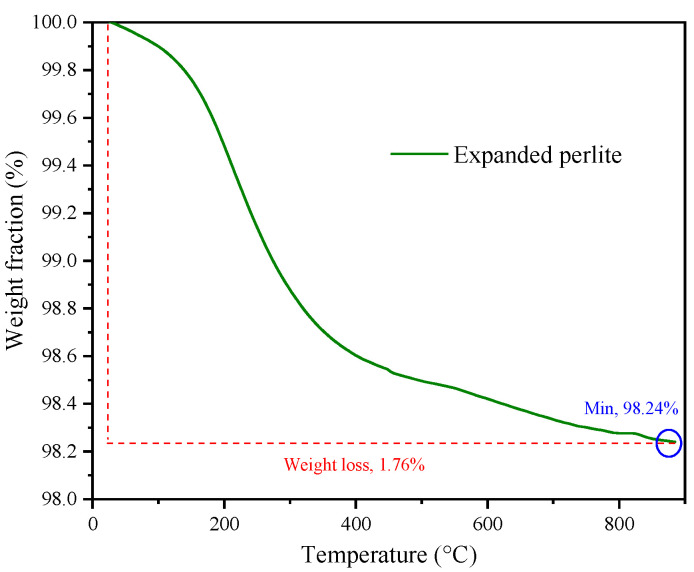
Weight loss of EP between 20–900 °C.

The EP used in the hot-mix asphalt mixture should possess good thermal stability. The weight loss of the EP in the range of 20 °C to 900 °C is shown in Figure 5. In Figure 5, the mass loss of the EP at 900 °C is only 1.76%, indicating that the EP has good thermal stability and can be used as filler for hot-mix asphalt mixtures.

**Figure 6:**
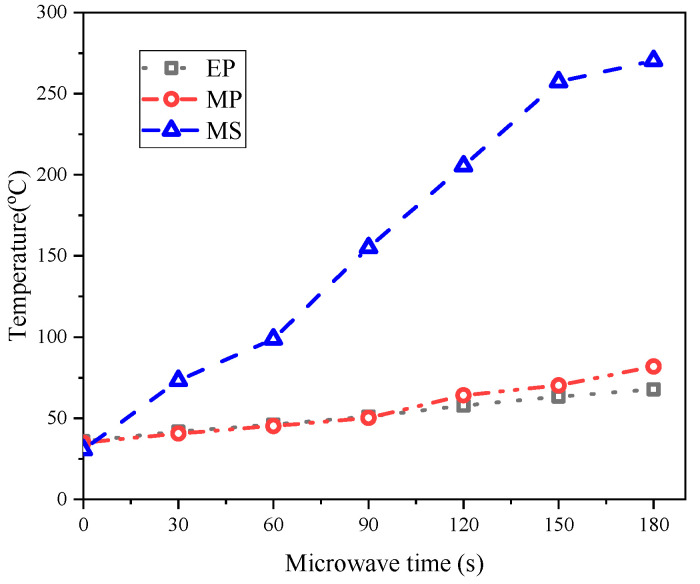
Heating efficiencies of EP, MP and MS powders after 180 s microwave radiation.

The microwave heating efficiencies of EP, MP and MS are shown in Figure 6. In Figure 6, the MS has excellent heat-generation capacity, while the EP has the lowest heat-generation capacity, which means that even if microwaves reach the underlying structure, microwave energy cannot be converted into heat energy and cause energy loss due to the EP. In addition, the metal content contained within the MS may enhance the thermal conductivity of the sample, and the rapid conduction of heat generated by the heating layer is what is exactly needed for the goal of rapid ice melting. Therefore, it is inferred from the two aspects of chemical composition and microwave heating efficiency that it is feasible to use the EP in the heat-resistance layer in the field of microwave de-icing.

### 3.2. Surface Heating Rates of Specimens

**Figure 7:**
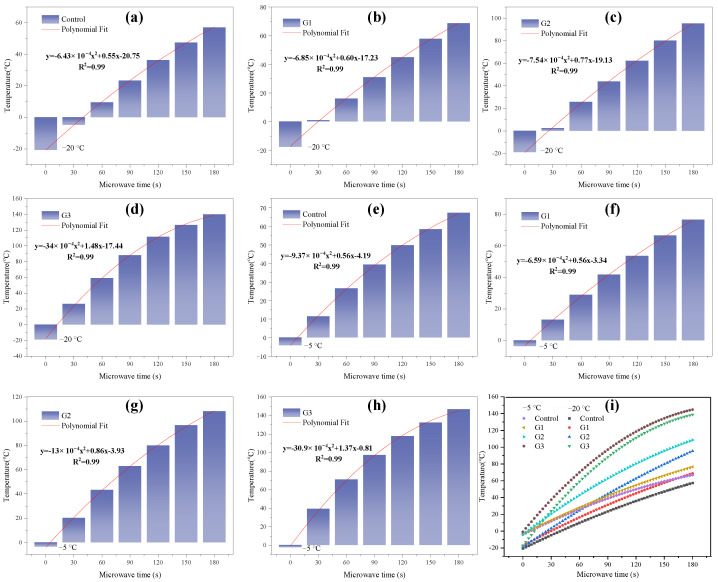
Average surface temperature changing with radiation time at different initial temperatures: (**a**) control, −20 °C; (**b**) G1, −20 °C; (**c**) G2, −20 °C; (**d**) G3, −20 °C; (**e**) control, −5 °C; (**f**) G1, −5 °C; (**g**) G2, −5 °C; (**h**) G3, −5 °C; and (**i**) summary.

The average surface temperatures of the specimens changing with radiation time at different initial temperatures are shown in Figure 7. In Figure 7, the correlation between the horizontal and vertical coordinates can be expressed by a quadratic function, and the regression coefficients all reach 0.99. HRS G3 shows excellent microwave heating efficiency. At −5 °C, the average heating rate of HRS G1, HRS G2 and HRS G3 at 180 s radiation time are 0.44 °C/s, 0.62 °C/s, 0.82 °C/s, respectively, which is 1.13, 1.61 and 2.17 times of the control group; this ratio increases to 1.20, 1.67, and 2.45 times at the initial temperature of −20 °C, respectively. It verifies that the structural layer optimization of the asphalt mixture can obtain a better heating rate regardless of the initial temperature.

The results of the study [26] have shown that the microwave of the receptor in the microwave radiation process is limited by its own shape, size and heat loss so that the heating rate can gradually decrease with an increase in the radiation time. In Figure 7i, the reduction in the heating rate of HRS G3 is the most obvious, although it possesses the most excellent microwave heating efficiency. This means that the continued heating ability of the asphalt mixture in the later stage can be weakened by reducing the heating layer’s thickness. The higher the temperature of the same heat dissipation area, the faster its heat loss; therefore, it is conducive for HRS G3 to melt the frozen sticky layer efficiently. In addition, the reduction in the thickness of the heating layer leads to a decrease in the content of absorbing heating materials such as MS, which affects the continuous heating capacity in the later period. However, this is not important for the fast road microwave de-icing process, and the pursuit of a faster early heating rate is the primary goal.

**Figure 8:**
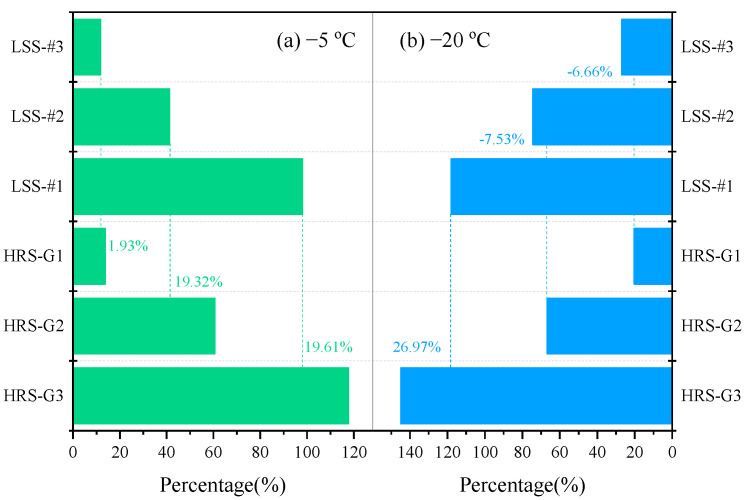
Average surface temperature rise rate comparison of specimens after 180 s microwave radiation at different initial temperatures: (**a**) −5 °C; (**b**) −20 °C.

The average surface temperature of the HRS and LSS groups were normalized according to Equation (1) to verify the effectiveness of the heat-resistance design, and the results are displayed in Figure 8.

(1) $$y=\frac{T_{i}-T_{control}}{T_{control}}$$

where *y* is the improvement ratio compared to the control group, *T_i_* is the average surface temperature of each experimental group after 180 s radiation, °C; and *T_control_* is the average surface temperature of the control group after 180 s radiation, °C.

In Figure 8, the microwave heating efficiency of the HRS group contains the heat-resistance layer and is basically better than that of the LSS group. HRS G1, HRS G2 and HRS G3 increased the heating efficiency of the corresponding LSS group by 1.93%, 19.32% and 19.61%, respectively, when the initial temperature was −5 °C; HRS G3 even increased the heating efficiency of the corresponding LSS group by 26.97% when the initial temperature was −20 °C. It shows that the heat-resistance layer does hinder the conduction and loss of heat generated by the upper heating layer to the lower layer, which is conducive to the accumulation of heat on the specimen’s surface and can further improve the de-icing efficiency.

### 3.3. Surface Thermal Analyses of Specimens

**Figure 9:**
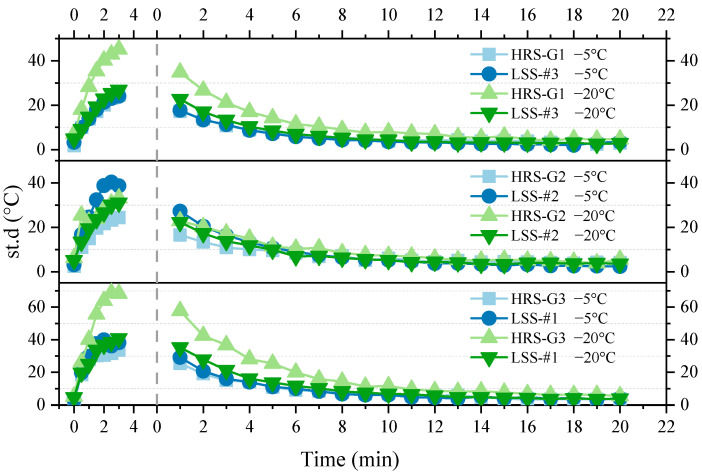
Standard deviations of the average surface temperature of HRS and LSS groups.

The standard deviation of each temperature point data in the pixel-level temperature map was taken as the criterion in this work. The size of the standard deviation is proportional to the severity of regional heat accumulation. The standard deviation of the average surface temperature of the specimen is presented in Figure 9. Each of the figures in Figure 6 contains two parts: one is the standard deviation of the average surface temperature during the heating process (0–3 min), and the other is the standard deviation during the cooling process (1–20 min) after microwave radiation.

A lower initial temperature can cause a higher standard deviation during both the heating process and cooling process. The standard deviation of the HRS groups rises as the heat-resistance layer thickness increases. This is due to the efficient heat-generation efficiency caused by the structural layer optimization. However, the average surface temperature’s standard deviation of all specimens basically reached a stable state when the microwave radiation process finished in 10 min, indicating that the heat on the surface had been evenly dispersed at this time. Therefore, we can consider it as separating the microwave heating process from the mechanical de-icing measures and thus setting an “empty window period” for heat conduction between them. In any case, the problem of how to quickly disperse the heat generated by the heating layer under low-temperature conditions still needs to be further solved.

**Figure 10:**
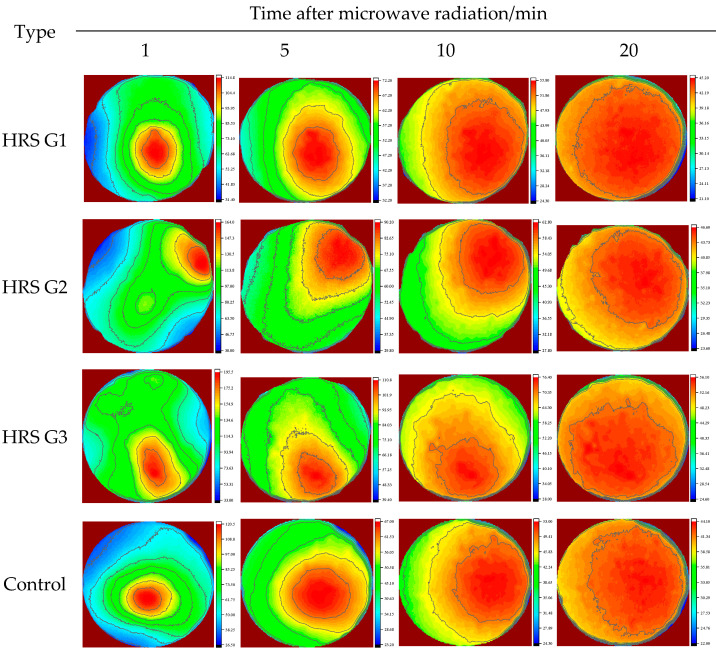
Heat diffusion on specimens’ surface after radiation.

**Figure 11:**
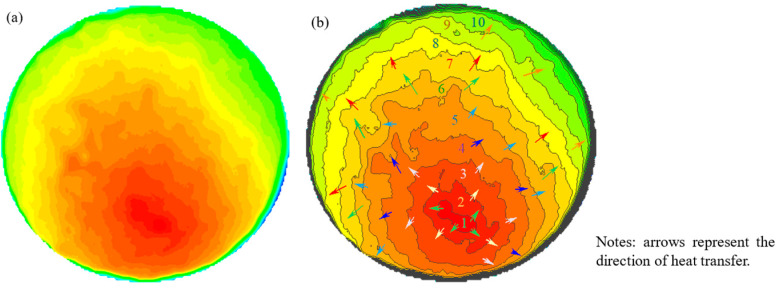
Circular corrugated heat diffusion form: (**a**) surface heat distribution of HRS G3; (**b**) contour plot of (**a**).

In addition, the surface heat-spread diagrams with radiation times of 1 min, 5 min, 10 min, and 20 min are shown in Figure 10. The heat distribution of all specimens was uneven, and the heat began to spread to the low-temperature region layer by layer as time went on. At about 10 min, the heat diffusion area exceeded 80%, and at 20 min, the heat had spread to the full surface. The average surface temperature of HRS G3 at 10 min after microwave radiation is shown in Figure 11. In Figure 11a, it is apparent that the heat spreads to the periphery in a layer-by-layer manner from the high-temperature zone. The average surface temperature of HRS G3 in Figure 11a was divided by a gradient of 10 °C, and the result is shown in Figure 11b. In Figure 11b, the entire surface was divided into more than 10 temperature intervals, which indicates that the surface heat is nucleated by the heat accumulation region and spreads like a water wave to the periphery.

### 3.4. Heat Conduction In-Depth Direction

**Figure 12:**
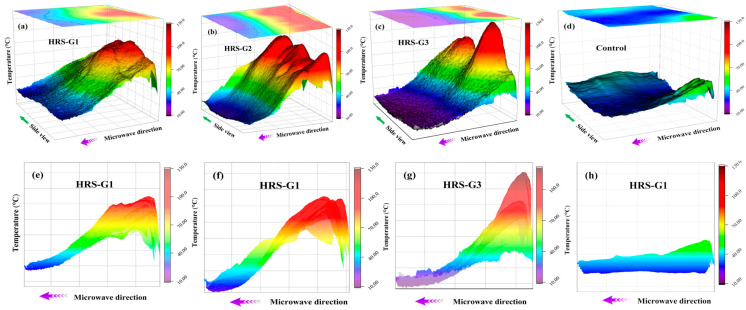
Microwave radiation depth after 180 s microwave radiation time: (**a**–**d**) are the oblique views of HRS G1, HRS G2, HRS G3 and control group, respectively; (**e**–**h**) are the side views of HRS G1, HRS G2, HRS G3 and control group, respectively.

The heat-resistance layer designed in this work is located in the lower structure of the asphalt mixture, and its main function is to prevent the heat generated by the upper heating layer from conducting downwards. Therefore, the radiation depths of different specimens after 180 s of microwave radiation were tested to detect the thermal-resistance effect. In Figure 12, the height and color of the heat peak represent the temperature. The starting point of the microwave direction is the specimen surface, and the endpoint is the bottom surface of the specimen. The temperature of each specimen showed an obvious downward trend from the surface to the bottom in Figure 12a–d. The peak heights of the control and the HRS G1–HRS G3 surfaces gradually increase in Figure 12e–h, indicating that the heat on the surface increases as the heat-resistance layer thickness increases.

**Figure 13:**
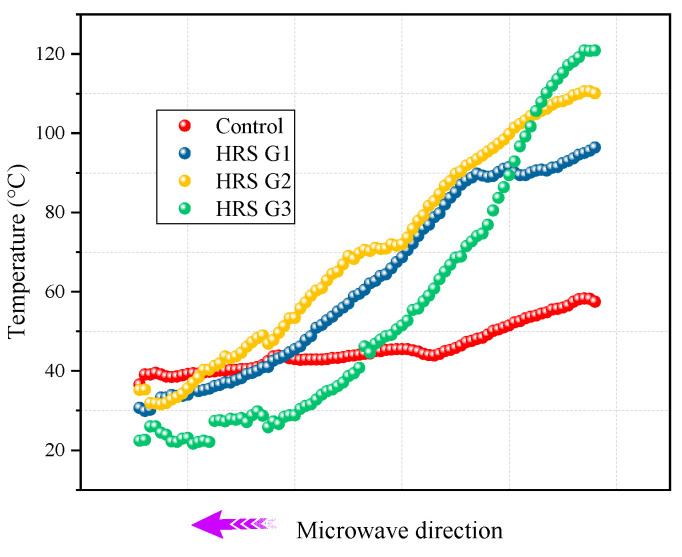
Maximum temperature of the side view of HRS groups.

The highest temperature corresponding to each abscissa in Figure 12e–h is extracted to illustrate the heat-resistance layer function, and the results are presented in Figure 13. In Figure 13, the temperature of the control group in the depth direction is steadily distributed, indicating that the upper and lower asphalt mixtures consume almost the same amount of microwave energy, resulting in a waste of energy. The temperatures of the upper and lower layers of each specimen in the HRS groups have obvious differences. For example, HRS G3 has the lowest lower temperature as well as the highest average surface temperature at the same time. There are two reasons for this situation. One is the structural layer optimization of the asphalt mixture, which increases the heat production rate. The other is that the introduction of the heat-resistance layer hinders the heat conduction to the lower layer, so more heat is gathered to the upper layer for de-icing.

**Figure 14:**
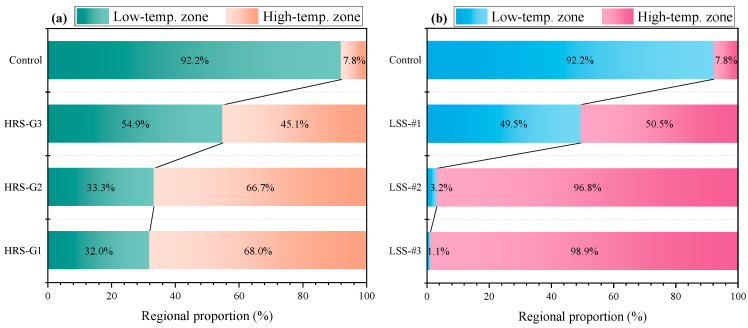
Calculation results of high- and low-temperature zone proportions with 40 °C as the limit: (**a**) HRS; (**b**) LSS.

The temperature distributions in the depth direction of the HRS and the LSS groups were compared to further clarify the role of the heat-resistance layer, and the results are represented in Figure 14. In Figure 12e–h, the temperature of each specimen is divided into a high-temperature zone and a low-temperature zone with a limit of 40 °C, as shown in Figure 14. The high-temperature zone contains the heating layer and part of the heat-resistance layer, and the low-temperature zone contains the remaining heat-resistance layer. The ratio of the low-temperature zone represents the thermal-resistance effect. The larger the low-temperature zone, the worse the thermal-resistance effect. In Figure 14, the overall temperature of the control group is lower, so the proportion of the low-temperature zone is larger. The ratio of the low-temperature zone of the specimens in the HRS and the LSS groups gradually increases with the decrease in the heating layer thickness, which highlights the effect of structural layer optimization. Comparing the HRS and the LSS groups with the same thickness of the heating layer, it is found that the ratio of the low-temperature zone of the HRS groups is substantially larger than that of the LSS groups, suggesting that the heat-resistance layer does hinder the downward conduction of heat.

### 3.5. De-Icing Time

**Figure 15:**
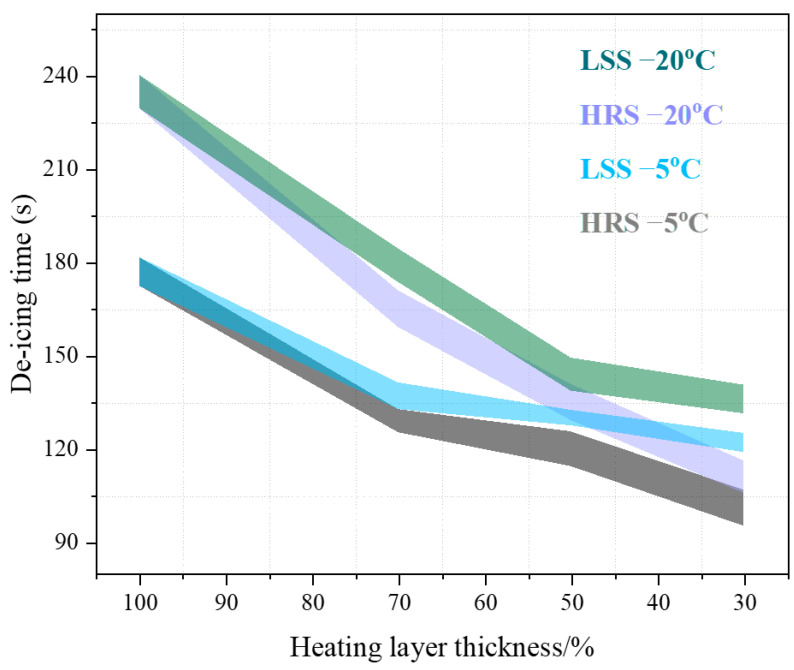
Relationship between de-icing time and heating layer thickness.

The de-icing times of the LSS and the HRS groups under different initial temperatures are shown in Figure 15. In Figure 15, the de-icing time continuously decreases as the thickness of the heating layer decreases. Taking HRS −20 °C as an example, the de-icing times of HRS G1, HRS G2 and HRS G3 were reduced by 29.79%, 42.55% and 52.77%, respectively, compared with the control group. In addition, the initial temperature also has a certain effect on the de-icing time. For HRS −5 °C, the de-icing times of HRS G1, HRS G2 and HRS G3 were reduced by 27.12%, 32.20% and 42.94%, respectively, compared with the control group, indicating that the lower the initial temperature, the more obvious the effect of structural layer optimization. Finally, the design of the heat-resistance layer can further reduce the de-icing time. For LSS −20 °C, the de-icing times of LSS #3, LSS #2 and LSS #1 were reduced by 23.83%, 38.72% and 42.13%, respectively, compared with the control group. It suggests that the heat-resistance layer can additionally increase the ice de-icing efficiency by up to 10%.

## 4. Discussions

### 4.1. Heat-Resistance Layer Function in Heat Transfer

The heat transfer inside the pavement structure is achieved through heat conduction. The asphalt mixture designed in this work is divided into two layers: a heating layer and a heat-resistance layer. The heating layer and the heat-resistance layer are regarded as two independent parts in contact with each other, and the heat generated by the heating layer is gradually transferred to the heat-resistance layer from top to bottom in the form of heat conduction. The more heat transferred to the heat-resistance layer, the greater the heat loss and the less conducive to the effective use of energy. The heat flow through the layer with an arbitrary thickness of *dx* in the *x* direction per unit of time, according to Fourier’s law [23], is

(2) $$\phi =-\lambda \frac{dT}{dx}A$$

where, ϕ is the heat transfer rate, W; *λ* is the thermal conductivity, W·m^−1^·K^−1^; *T* is the temperature, K; and *A* is the cross-sectional area of heat transfer, m^2^.

**Figure 16:**
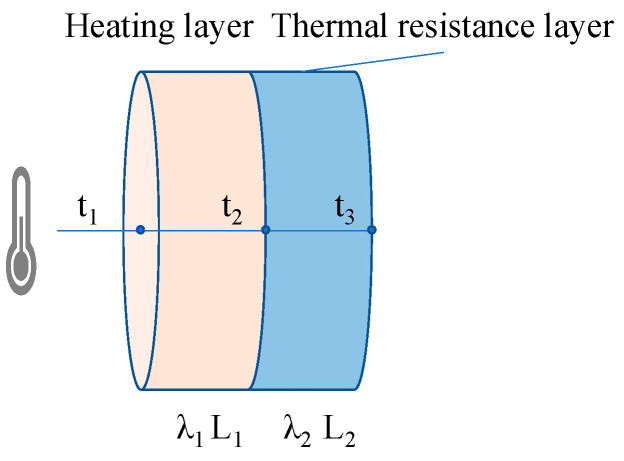
Internal heat transfer process of asphalt mixture.

The internal heat transfer process model inside the mixtures is constructed under the following assumptions: (1) the heat transmits along one direction, i.e., the temperature gradient only exists in one dimension; (2) the external ambient temperature and humidity are not taken into account; and (3) the distribution of aggregates and voids in the mixtures is not taken into account. The internal heat transfer process of the mixtures is displayed in Figure 16. In Figure 16, the heating layer and the heat-resistance layer have a common surface area A, and the temperatures of the upper surface layer, the intermediate layer, and the lower surface layer of the heat-resistance layer are t_1_, t_2_ and t_3_, respectively; the thermal conductivity of the heating layer and the heat-resistance layer are different as a result of the differences in their material composition. The following two parts of the heat-flow calculation formulas can be obtained based on Equation (2).

(3) $$\phi =-\frac{\lambda_{1}A}{L_{1}}\left(t_{2}-t_{1}\right)$$

(4) $$\phi =-\frac{\lambda_{2}A}{L_{2}}\left(t_{3}-t_{2}\right)$$

(5) $$L_{1}+L_{2}=0.635$$

Combine Equations (3) and (4) and give Equation (6)

(6) $$\phi =-A\left(t_{3}-t_{1}\right)/\left(\frac{L_{1}}{\lambda_{1}}+\frac{L_{2}}{\lambda_{2}}\right)$$

It can also be expressed as

(7) $$\phi =AK\left(t_{1}-t_{3}\right)$$

where

(8) $$K=1/\left(\frac{L_{1}}{\lambda_{1}}+\frac{L_{2}}{\lambda_{2}}\right)$$

Transform Equation (8) and obtain Equation (9),

(9) $$\frac{1}{AK}=\frac{L_{1}}{A\lambda_{1}}+\frac{L_{2}}{A\lambda_{2}}$$

**Figure 17:**
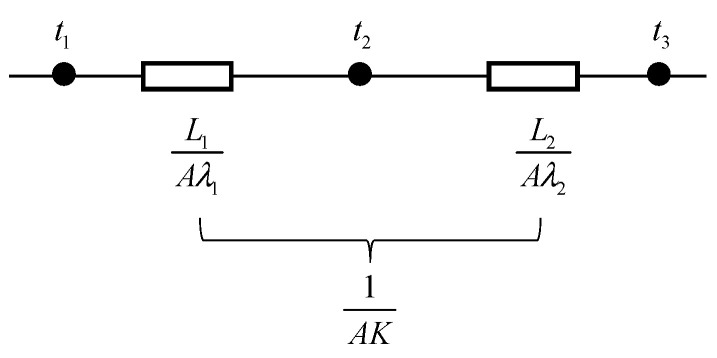
Thermal-resistance analyses during heat transfer.

1/*AK* can be defined as the overall thermal resistance in the heat transfer process by analogy to Ohm’s law; *L*_1_/*Aλ*_1_ and *L*_2_/*Aλ*_2_ are the thermal resistances of the heating layer and the heat-resistance layer, respectively, and the two together constitute the total thermal resistance of the asphalt mixture, as shown in Figure 17.

According to Equation (5), we can obtain

(10) $$\frac{1}{AK}=\frac{0.635\lambda_{1}+\left(\lambda_{2}-\lambda_{1}\right)L_{1}}{A\lambda_{1}\lambda_{2}}$$

In Equation (10), the thermal conductivity of each layer is not related to the layer’s thickness. The incorporation of EP can undoubtedly make the thermal conductivity of the heat-resistance layer lower than that of the heating layer according to the above research results. The thermal conductivity of the lower asphalt mixture of the samples with the same structural design in the LSS group is higher than that of the HRS group, so its total thermal resistance is lower. The thermal conductivity of all of the heat-resistance layers and the heating layers in each HRS group specimen remains constant when the content of the EP is fixed. At this time, the total thermal resistance of the asphalt mixture is only determined by the thickness of the heating layer according to Equation (10). The total thermal resistance gradually increases as the thickness of the heating layer decreases. HRS G3 has the lowest heating layer thickness and the largest thermal-resistance value, which is beneficial for reducing the heat loss rate and improving energy utilization.

### 4.2. Cost-Effectiveness Analyses

The microwave heating rate and ice de-icing efficiency of asphalt pavement can be enhanced by the design methods, regardless of the type and content of the microwave-absorbing materials. Table 4 displays the differences in raw material costs of several groups of asphalt mixtures without considering the transportation costs, differences in paving processes, or environmental factors.

In Table 4, the raw material cost of the fully filled microwave de-icing pavement, designed by the traditional scheme, is increased by 33.4% compared with the ordinary asphalt pavement, while structural layer optimization reduces the raw material cost by up to 17.5% (LSS #1) compared to the traditional design scheme, and at the same time, increased the microwave heating efficiency by 118.0% (−20 °C), as shown in Section 3.2. In addition, the heat-resistance design of the lower structural layer can increase the raw material cost of the corresponding LSS group by 13.0% (HRS G3) at the minimum, but the microwave heating efficiency can be further enhanced by 27.0%. The improvement in microwave heating efficiency is attributed to the improvement in heat-generation efficiency brought about by the optimization of the asphalt mixture’s structure’s layer and the function of reducing energy dissipation by the thermal-resistance layer.

Therefore, the collaborative design method of structural layer optimization and heat-resistance design can increase energy efficiency and greatly save microwave-absorbing materials incorporated in asphalt pavements while ensuring excellent microwave de-icing efficiency.

## 5. Conclusions

To eliminate driving safety hazards caused by winter icing on asphalt pavements, the microwave heating and de-icing efficiency of asphalt mixtures containing MS and EP were evaluated. The following conclusions were drawn.

(1) The microwave heating efficiency of specimens via structural layer optimization can be further improved by up to 26.97%, ascribed to the extra heat-resistance design. The heat-resistance layer can prevent the heat generated by the upper heating layer from being conducted and lost to the lower layer, as well as promote the heat to concentrate on the specimen’s surface.

(2) The heat-resistance layer can promote the uniform dispersion of heat when the initial temperature is high. It is recommended to set an “empty window period” for heat conduction between the microwave heating process and the mechanical de-icing measures.

(3) The microwave de-icing efficiency is positively related to the heat-resistance layer’s thickness and initial temperature while negatively related to the heating layer’s thickness. The de-icing time can be sharpened more than twice by optimizing the structure of the asphalt mixture and can be further reduced by 10% via the heat-resistance layer.

(4) The total thermal resistance of the asphalt mixture can be increased by reducing the thickness of the heating layer and incorporating expanded perlite powder. The collaborative design method of structural layer optimization and heat-resistance design enhanced energy efficiency and greatly saved the microwave-absorbing materials used in asphalt pavements while ensuring excellent microwave de-icing efficiency.

## 6. Further Study

Future research will focus on the use of in situ testing tools to focus on the effects of the thermal properties of raw materials on the asphalt mixture’s key indicators, such as the mixture structure, microwave heating properties and mechanical properties.

## Figures and Tables

**Table 1:** Properties of aggregates.

Aggregates	Bulk Density (g/cm^3^)	Water Absorption (%)	Needle Flake Content (%)	Crushing Value (%)	Los Angeles Abrasion (%)
Magnetite slag	3.418	0.67	10.3	13.5	21.2
Limestone	2.836	0.30	11.2	15.6	23.5

**Table 2:** Properties of asphalt.

Properties	Results
Penetration (25 °C, 5 s, 100 g; 0.1 mm)	93.5
Softening Point (°C)	48.3
Ductility (15 °C, 5 cm/min; cm)	63
Solubility (%)	99.5
Density (15 °C, g/cm^3^)	1.03
Wax content (distillation method, %)	1.6
Flash Point (°C)	264

**Table 3:** Mixing proportion for Marshall specimens.

Structural Design of Marshall Specimens		Sieve Size (mm)													Asphalt/Aggregate Ratio (%)
		16	13.2	9.5	4.75		2.36	1.18	0.6	0.3	0.15	0.075	Filler		
					LS	MS							MP	EP	
HRS G1	Heating layer	0	41.8	154.6	0	236.6	133.7	87.8	62.7	46.0	29.3	33.4	50.1	0	4.5
	Heat-resistance layer	0	17.9	66.3	84.2	0	57.3	37.6	26.9	19.7	12.5	14.3	0	18.3	4.7
HRS G2	Heating layer	0	29.8	110.4	0	169.0	95.5	62.7	44.8	32.8	20.9	23.9	35.8	0	4.5
	Heat-resistance layer	0	29.8	110.4	140.3	0	95.5	62.7	44.8	32.8	20.9	23.9	0	30.4	4.7
HRS G3	Heating layer	0	17.9	66.3	0	101.4	57.3	37.6	26.9	19.7	12.5	14.3	21.5	0	4.5
	Heat-resistance layer	0	41.8	154.6	196.4	0	133.7	87.8	62.7	46.0	29.3	33.4	0	42.6	4.7
Control	One layer	0	59.7	220.9	0	338.1	191.0	125.4	89.5	65.7	41.8	47.8	71.6	0	4.5

Note: LS is limestone; MS is magnetite slag; MP is mineral powder; EP is the expanded perlite powder.

**Table 4:** Normalized raw material costs of microwave de-icing pavement.

Pavement Type	Normalized Cost	Pavement Type	Normalized Cost
Plain asphalt mixture	1.000	Plain asphalt mixture	1.000
Control	1.334	Control	1.334
LSS #3	1.234	HRS G1	1.419
LSS #2	1.167	HRS G2	1.323
LSS #1	1.100	HRS G3	1.230

Note: Plain asphalt mixture represents an ordinary pavement without wave-absorbing materials; the control group represents a fully filled microwave de-icing pavement. The mass substitution ratio of limestone aggregate and magnetite in the asphalt mixture is 1:1.21, while it is 1:0.85 for the MP and EP due to the difference in bulk densities. The ratio of the price per kilogram of limestone to magnetite is 1:2, while it is 1:3 for the MP and EP according to the price in the Chinese market (May 2024).

## Data Availability

The original contributions presented in the study are included in the article, further inquiries can be directed to the corresponding author.
